# Do clinical and communication skills scores on credentialing exams predict potentially inappropriate antibiotic prescribing?

**DOI:** 10.1186/s12909-023-04817-w

**Published:** 2023-11-01

**Authors:** Robyn Tamblyn, Teresa Moraga, Nadyne Girard, John Boulet, Fiona K. I. Chan, Bettina Habib

**Affiliations:** 1 Department of Epidemiology, Biostatistics and Occupational Health, McGill University, 2001 McGill College Avenue, H3A 1G1 Montreal, QC Canada; 2 Department of Medicine, McGill University Health Center, Montreal, QC Canada; 3 Clinical and Health Informatics Research Group, McGill University, Montreal, QC Canada; 4 Foundation for Advancement of International Medical Education and Research (FAIMER), Philadelphia, PA USA

**Keywords:** Antibiotic prescribing, Communication skills, Clinical skills, Credentialing examination, OSCE

## Abstract

**Background:**

There is considerable variation among physicians in inappropriate antibiotic prescribing, which is hypothesized to be attributable to diagnostic uncertainty and ineffective communication. The objective of this study was to evaluate whether clinical and communication skills are associated with antibiotic prescribing for upper respiratory infections and sinusitis.

**Methods:**

A cohort study of 2,526 international medical graduates and 48,394 U.S. Medicare patients diagnosed by study physicians with an upper respiratory infection or sinusitis between July 2014 and November 2015 was conducted. Clinical and communication skills were measured by scores achieved on the Clinical Skills Assessment examination administered by the Educational Commission for Foreign Medical Graduates (ECFMG) as a requirement for entry into U.S residency programs. Medicare Part D data were used to determine whether patients were dispensed an antibiotic following an outpatient evaluation and management visit with the study physician. Physician age, sex, specialty and practice region were retrieved from the ECFMG databased and American Medical Association (AMA) Masterfile. Multivariate GEE logistic regression was used to evaluate the association between clinical and communication skills and antibiotic prescribing, adjusting for other physician and patient characteristics.

**Results:**

Physicians prescribed an antibiotic in 71.1% of encounters in which a patient was diagnosed with sinusitis, and 50.5% of encounters for upper respiratory infections. Better interpersonal skills scores were associated with a significant reduction in the odds of antibiotic prescribing (OR per score decile 0.93, 95% CI 0.87–0.99), while greater proficiency in clinical skills and English proficiency were not. Female physicians, those practicing internal medicine compared to family medicine, those with citizenship from the US compared to all other countries, and those practicing in southern of the US were also more likely to prescribe potentially unnecessary antibiotics.

**Conclusions:**

Based on this study, physicians with better interpersonal skills are less likely to prescribe antibiotics for acute sinusitis and upper respiratory infections. Future research should examine whether tailored interpersonal skills training to help physicians manage patient expectations for antibiotics could reduce unnecessary antibiotic prescribing.

**Supplementary Information:**

The online version contains supplementary material available at 10.1186/s12909-023-04817-w.

Overuse of antibiotics is a worldwide challenge and an important driver of antimicrobial resistance. Antibiotics are often prescribed for conditions that are predominantly of viral etiology, particularly upper respiratory infections and sinusitis [1–3]. Almost all studies have reported considerable variation in the likelihood of antibiotic prescribing between physicians [4–8]. It is hypothesized that diagnostic uncertainty, aversion to risks related to errors of omission, and a bias towards action versus inaction may contribute to unnecessary antibiotic prescribing [9]. A novel study of blinded standardized patient visits in China supports this hypothesis. Physicians were more likely to prescribe antibiotics for conditions of non-bacterial origin if they had poorer diagnostic ability, which in turn was related to the quality of their history and physical examination [10].

Ineffective communication between physicians and patients, particularly in relationship to patient demand for treatment is also considered to be an important determinant of antibiotic overuse. Both public health campaigns aimed at reducing patient demand through antibiotic education, and targeted communication training for physicians have been effective in reducing antibiotic prescribing for conditions that are mainly of viral etiology [11–15].

The importance of effective clinical and communication skills in clinical practice has been recognized by medical educators and credentialing bodies. It is also acknowledged that written examinations are unable to adequately assess clinical and communication skills [16, 17]. Moreover, the perceived decline in rigorous clinical skills training in medical education was recognized as an important gap in preparing qualified graduates [18–20]. The invention of the objective structured clinical examination (OSCE) provided a rigorous approach to the assessment of clinical and communication skills in a simulated clinical environment and was rapidly implemented in both undergraduate and postgraduate health professional training programs [21]. This format of assessment was also adopted by credentialing bodies, tasked with the obligation of determining the safety and effectiveness of medical trainees to enter postgraduate training and medical practice. The Educational Commission for Foreign Medical Graduates (ECFMG) was the first credentialing body to introduce the OSCE examination as a requirement for international medical graduates to enter postgraduate training in the United States [22]. International medical graduates had been identified as physicians who were more likely to prescribe potentially unnecessary antibiotics in some but not all studies [4, 23–25]. The introduction by the ECFMG of the clinical skills assessment OSCE in 1988 provided the first opportunity to evaluate whether clinical and communication skills are associated with potentially unnecessary antibiotic prescribing for upper respiratory infections and sinusitis after 8 to 15 years in practice.

## Methods

### Design and population

A retrospective cohort study of international medical graduates was conducted. Physicians were eligible for inclusion in the cohort if they successfully completed the Educational Commission for Foreign Medical Graduates (ECFMG) clinical assessment OSCE examination, entered practice in the United States in primary care (family medicine, general practice, internal medicine), and conducted an evaluation and management visit for a Medicare patient whom they diagnosed as having an upper respiratory infection or sinusitis. All physicians who completed the required ECFMG clinical assessment examination were first identified. Physicians were then linked by first and last name, sex, and birthdate to the American Medical Association (AMA) Masterfile to identify those who had acquired a license to practice in the United States. The AMA national provider identifier of each physician was used to link to the Center for Medicare and Medicaid Services (CMS) administrative files to identify physicians in the cohort who had billed for Medicare patients. All patients seen by these physicians in 2014 and 2015 were identified in the Medicare Carrier RIF file, inpatient files, outpatient file, and Part D files and then all health care services and medications received by these patients by any health professional were retrieved. As earlier studies have shown associations between clinical competence, communication ability and quality of care indicators after 3 to 7 years in practice [26–29], we aimed to determine if these associations would persist, even after physicians had been in practice 8 to 15 years.

Patients were eligible for consideration if they were enrolled in Medicare, had an evaluation and management visit with a study physician in an outpatient setting between July 2014 and November 2015 with a diagnosis of upper respiratory infection or acute sinusitis (Appendix 1 for ICD codes), had continuous Part D drug coverage, had not been diagnosed with chronic sinusitis in the 6 months prior to the evaluation visit, and did not have an active supply of an antibiotic prescription on the date of the visit. The start date of follow-up allowed us to obtain at least 6 months of baseline information about patient characteristics before the evaluation visit for sinusitis or URI with the study physician. The end date allowed us a 30 day follow-up period to evaluate whether an antibiotic was prescribed/dispensed after the evaluation visit. If patients had visits for both upper respiratory infection and sinusitis, the first visit for either condition was selected.

### Outcome: antibiotic prescription

The dispensation of an antibiotic was measured in the 30 days following the evaluation and management visit to the study physician using the Part D drug insurance file. Antibiotic drugs included the penicillins, cephalosporins, tetracyclines, macrolides, clindamycin, sulfonamides, quinolones, and metronidazole. The prescribing physician had to be the study physician who conducted the evaluation visit.

### Predictors

#### Clinical skills and communication ability

Scores from the Clinical Skills Assessment (CSA) OSCE Examination between 1998 and 2004 were used to measure clinical skills and communication ability. The CSA consisted of 10 or 11 modeled encounters between the candidate and a standardized patient. An overall *clinical skills score* was given based on history taking and physical examination conducted in these encounters and each candidate’s diagnosis and management plan as written in a post-encounter clinical note. An overall *communication score* was given based on the candidate’s interpersonal skills, assessed in each encounter by the standardized patient, as well as their spoken English proficiency. An acceptable clinical skills and communication score was required to pass the examination. The reliability for the various test components ranges from 0.71 to 0.82 [30]. The CSA was put in place to ensure that all IMGs could demonstrate an acceptable level of clinical skills necessary for entry into US graduate medical education programs. The CSA was subsequently replaced by USMLE Step 2 Clinical Skills, which, as of 2004, was required for graduates of all US and foreign medical schools, up until the start of the pandemic [31].

#### Other physician characteristics

Physician age, sex, specialty, and practice region have been associated with a variety of quality of care indicators [32–36]. These data were retrieved from the ECFMG database and the AMA Masterfile. As the type and rate of infections varies between different countries, we hypothesized that the physician’s country of origin may influence the likelihood of antibiotic prescribing. Physician citizenship at the time of medical school graduation was obtained from the ECFMG database and grouped into twelve geographic regions.

#### Patient characteristics

Patient characteristics that could influence the likelihood of antibiotic prescribing may differ between physician practices. For this reason, we measured patient sex, and race (White, Black, Asian, Hispanic, NA Native, other) using data from the CMS Master Beneficiary Summary File. Multimorbidity has been found to increase the likelihood antibiotic drug prescribing. We used the Elixhauser index [37, 38] to measure co-morbidities using diagnostic data from the outpatient, inpatient and carrier files in the six months prior to the evaluation and management visit. A count of the number of active medications at the time of the evaluation and management visit was also measured using the Part D files.

### Analysis

Descriptive statistics were used to summarize physician and patient characteristics. To estimate the association between clinical skills, communication ability and the odds of antibiotic prescribing for primary and secondary prevention, we used GEE logistic regression. Patient was the unit of analysis and physician was the clustering factor, accounted for using an exchangeable correlation coefficient. The component sub-scores for clinical skills (history and physical examination, diagnosis and management) and communication (interpersonal skills and English proficiency) were fit in separate models as the adequacy of history and physical examination, diagnostic ability, and interpersonal skill proficiency have all been shown to be associated with antibiotic prescribing in prior studies [10, 15, 26]. Each score was treated as a continuous variable, and models were adjusted for other physician and patient characteristics. There may be a non-linear association between clinical and communication skills scores and antibiotic prescribing, possibly a minimum threshold of skills needed to manage these encounters. To assess for non-linear effects, we included a score squared term in the model and used the Wald Chi-square to assess significance. All analyses were done using SAS version 9.4.

## Results

A total of 2,526 physicians were included in the cohort, 54.8% of whom were male with a mean age of 43.8 years (Table 1). The majority were citizens of India (25.7%), United States (21.1%), or Asia/Oceania (13.8%), 58.3% specialized in family medicine, and 37.7% practiced in the south of the United States. With respect to clinical skills, physicians scored higher on their skill in conducting a history and physical examination (mean 67.8%) compared to diagnosis and management (mean: 59.3%). For communication, English proficiency scores (mean 86.0%) were higher than interpersonal skills (mean 76.6%).

**Table 1:** Characteristics of the 2,526 International Medical Graduates

Physician Characteristics	
	**Mean (SD)**
**Age**	43.82 (5.50)
**Gender**	**N (%)**
Female	1141 (45.17%)
Male	1385 (54.83%)
**Citizenship**	
Africa	145 (5.74%)
Canada	27 (1.07%)
Eastern Europe	182 (7.21%)
Europe	62 (2.45%)
India	650 (25.73%)
Mexico/Central America/Caribbean	111 (4.39%)
Middle East	200 (7.92%)
Oceania/Asia	349 (13.82%)
Pakistan	158 (6.25%)
South America	75 (2.97%)
United Kingdom	31 (1.23%)
United States	536 (21.22%)
**Specialty**	
Family Medicine	1472 (58.27%)
Internal medicine	1054 (41.73%)
**Region of Practice**	
Northeast	552 (21.85%)
Midwest	539 (21.34%)
South	952 (37.69%)
West	483 (19.12%)
**Clinical Skills Examination Scores**	**Mean (SD)**
**Clinical Skills**	
History and physical examination score	67.77 (6.65)
Diagnosis and management score	59.32 (9.50)
**Communication Skills**	
English proficiency score	86.02 (13.98)
Interpersonal skills score	76.60 (7.76)

Overall, 48,394 patients were eligible for inclusion, 66.5% were female, 81.2% were white, with a mean age of 68.9 years (Table 1). The presenting problem was diagnosed as upper respiratory infection (URI) in 56.5% of encounters and acute sinusitis in 43.5%. Most patients had co-morbid conditions, with 34.8% having five or more conditions, and 33.6% were taking six or more prescribed medications. In 59.5% of encounters, an antibiotic was prescribed, in 71.1% of encounters for sinusitis and in 50.5% for upper respiratory infections. The most common antibiotics prescribed were azithromycin (URI 53.8%; sinusitis 33.3%) and amoxicillin (URI 21.2%; sinusitis 38.5%).

Greater proficiency in history and physical examination (OR 0.97) and diagnosis and management (OR 0.99) was associated with a non-significant reduction in the odds of antibiotic prescribing (Table 2). While there was no association between English proficiency and antibiotic prescribing, interpersonal skills scores were associated with a significant reduction in the odds of antibiotic prescribing. For every decile increase in score, the odds of antibiotic prescribing were reduced by 7% (OR 0.93, 95% CI 0.87–0.99). This translates into a probability of receiving an antibiotic prescription of 57% for a physician who scores 62% in interpersonal skills and 49% for a physician scoring 92%; respectively two standard deviations below and above the mean interpersonal skills score. There was no evidence of a non-linear relationship.

**Table 2:** Characteristics of the 48,394 Medicare Patients with Upper Respiratory Infection or Acute Sinusitis

Patient Characteristics	
**Gender**	**N (%)**
Male	16,191 (33.46%)
Female	32,203 (66.54%)
**Age**	
18–64	11,066 (22.87%)
65–69	11,133 (23.00%)
70–75	11,915 (24.62%)
75+	14,280 (29.51%)
*Mean Age (SD)*	*68.9 (13.38)*
**Race**	
White	39,315 (81.24%)
Black	4658 (9.63%)
Asian	1202 (2.48%)
Hispanic	1896 (3.92%)
North American Native	150 (0.31%)
Other race	1173 (2.42%)
**Comorbidity**	
**Elixhauser Index**	
0 comorbidities	8528 (17.62%)
1–2 comorbidities	10,618 (21.94%)
3–4 comorbidities	12,395 (25.61%)
5 + comorbidities	16,853 (34.82%)
*Mean Elixhauser (SD)*	*3.79 (3.20)*
**Number of Prescribed Medications**	
0 active drugs	5448 (11.26%)
1–3 active drugs	16,261 (33.60%)
4–5 active drugs	10,427 (21.55%)
+ 6 active drugs	16,258 (33.60%)
*Mean Number Medications (SD)*	*4.62 (3.79)*
**Presenting Problem**	
Acute Sinusitis	21,068 (43.53%)
Upper Respiratory Infection	27,326 (56.47%)
**Antibiotic Prescribed**	
Yes	28,793 (59.50%)
No	19,601 (40.50%)

**Table 3:** Association between physician and patient characteristics and antibiotic prescribing

Physician Characteristics	OR 95%CI	P-value
**Clinical & Communication Skills Scores** ***(per decile increase)***		
**Clinical Skills**	0.98 (0.89; 1.07)	0.62
History and physical examination score	0.97 (0.90; 1.05)	0.48
Diagnosis and management score	0.99 (0.94; 1.04)	0.58
**Communication Skills**		
English proficiency score	1.00 (0.96; 1.04)	0.89
Interpersonal skills score	0.93 (0.87; 0.99)	0.02
**Physician Gender**		
Female	1.14 (1.02; 1.26)	0.02
Male	Ref	Ref
**Physician Age** (***per 5 year increase***)	1.02 (0.98; 1.01)	0.31
**Citizenship**		
Africa	0.71 (0.57; 0.88)	0.002
Canada	0.76 (0.51; 1.12)	0.16
Eastern Europe	0.88 (0.72; 1.08)	0.23
Europe	0.82 (0.59; 1.15)	0.26
India	0.89 (0.76; 1.03)	0.11
Mexico/Central America/Caribbean	0.49 (0.38; 0.63)	< 0.0.0001
Middle East	0.84 (0.69; 1.03)	0.10
Oceania/Asia	0.85 (0.72; 1.01)	0.06
Pakistan	0.89 (0.71; 1.11)	0.30
South America	0.67 (0.51; 0.87)	0.003
United Kingdom	0.69 (0.44; 1.06)	0.09
United States	Ref	Ref
**Physician Specialty**		
Primary care	0.81 (0.74; 0.90)	< 0.0.0001
Internal medicine	Ref	Ref
**Region of Practice**		
Northeast	Ref	Ref
Midwest	1.07 (0.93; 1.22)	0.37
South	1.34 (1.17; 1.52)	< 0.0001
West	0.83 (0.72; 0.96)	0.01
**Patient Characteristics**		
**Patient Age**	1.01 (1.00; 1.01)	0.11
**Patient Gender**		
Female	1.00 (0.96; 1.04)	0.98
Male	Ref	Ref
**Patient Race**		
White	Ref	Ref
Black	0.93 (0.87; 1.00)	0.04
Asian	1.04 (0.91; 1.18)	0.59
Hispanic	0.98 (0.89; 1.08)	0.66
North American Native	0.88 (0.67; 1.14)	0.32
Other race	1.04 (0.93; 1.17)	0.45
**Co-Morbidity**		
Elixhauser Index	0.99 (0.98; 1.00)	0.001
Number of Prescribed Medications	1.03 (1.02; 1.04)	< 0.0.0001
**Presenting Problem**		
Acute Sinus	2.76 (2.52; 3.02)	< 0.0.0001
Upper Respiratory Infection	Ref	Ref

After adjusting for interpersonal skills and other physician and patient characteristics, female physicians were 14% (OR 1.14, 95% CI 1.03–1.26) more likely to prescribe antibiotics compared to males (Table 2). Compared to physicians who were citizens of the United States at the time they completed medical school, physicians from all other countries were less likely to prescribe antibiotics, differences that were significant for Africa (OR 0.71, 95% CI 0.57–0.88), Central America (OR 0.49, 95% CI 0.38–0.63), and South America (OR 0.67, 95% CI 0.51–0.87). Physicians specializing in family medicine were 19% (OR 0.81, 95% CI 0.74–0.90) less likely to prescribe antibiotics compared to internal medicine specialists, and those practicing in the south and west regions of the United States were more and less likely to prescribe compared to the northeast, respectively. Patients were more likely to receive an antibiotic if they presented with acute sinusitis (OR 2.76, 95% CI 2.52–3.02) compared to upper respiratory infection, and were using a greater number of medications. They were less likely to receive an antibiotic if they were black compared to white and had higher levels of co-morbidity.

## Discussion

The ECFMG was the first agency in the world to introduce a standardized assessment of clinical and communication skills as part of the credentialing process. We found that physicians with higher scores for interpersonal skills on this examination were less likely to prescribe antibiotics for upper respiratory infection and acute sinusitis, even after 8 to 15 years in practice. Greater proficiency in history and physical examination, as well as diagnosis and management on the examination, were not significantly associated with antibiotic prescribing. Female physicians, internal medicine specialists, and those practicing in the south regions of the United States were also more likely to prescribe antibiotics. Physicians who were citizens of South or Central America were also less likely to prescribe compared to US citizens.

Although better interpersonal skills were associated with lower odds of antibiotic prescribing, better clinical skills were not. There may be several reasons for these differences in findings. First, this examination is taken before entering U.S residency programs that are designed to improve proficiency in data collection as well as diagnosis and management skills [39]. Thus, measurement of these clinical skills at the time of entry into residency may not reflect abilities 8 to 15 years after starting clinical practice. Second, while communications training has been shown to improve skills for some individuals, it may represent a more stable skill set [40] that is less amenable to sustained improvement with training. Indeed, interpersonal skills assessment has been introduced as part of the admissions process by some medical schools in order to select applicants who excel in this area. While the ECFMG was the first agency to introduce the OSCE as part of the credentialing process, it has since been implemented as a requirement for licensure in both Canada and the United States [16, 21]. Communication skills measured in the Canadian OSCE credentialing examination have been shown to predict the frequency of complaints to medical licensing authorities in the first 5 to 7 years in practice [27]. Other studies have found that the quality of a physician’s interpersonal skills increases patient satisfaction and adherence to treatment [41] as well as health outcomes [42–44]. Combined, these findings suggest that there may be benefits in measuring proficiency in interpersonal skills at medical school admission and on credentialing examinations because of their importance in optimizing the quality of clinical practice.

There are several mechanisms by which better communication skills may reduce the likelihood of antibiotic prescribing. First, physicians may be more likely to engage in patient education about the differences between viral and bacterial infections and their treatment [15]. Second, they may be more likely to address patient’s concerns about the severity of their symptoms and effective treatment [15, 45]. Failure to address patient concerns has been shown to increase the likelihood of antibiotic prescribing [45, 46]. Third, physicians with better communication skills may be more effective at addressing patient demand for antibiotics, using such strategies as online commentary during the physical examination about normal findings, the provision of contingency plans, and patient empowerment [45–47]. Indeed intervention studies to improve physician communication in managing patient expectations has been shown to reduce antibiotic prescribing [14, 48, 49].

The clinical and communication skills assessment component of both the United States Medical Licensure Examination (USMLE) and the Canadian Medical Council Examination was interrupted during the pandemic. The USLME Step 2 CS was subsequently permanently cancelled in 2021. Opponents to the examination pointed to the high cost for students who needed to travel to test centres for the examination, the existence of performance-based assessment of clinical and communication skills using OSCE -like examinations in most North American medical schools [50], and the need for the examination given an overall pass rate of over 90% [51]. Others have pointed to the adverse effects of eliminating the examination for international medical graduates and physicians from allopathic and osteopathic medical schools [52]. Regardless of the outcome, there is consensus that performance based assessment of clinical and communication skills is important and new methods of assessment are needed whether that be imbedded in accreditation processes of medical schools or as a component of licensing examinations.

Our findings are consistent with other studies that have shown that internal medicine specialists are more likely to prescribe antibiotics than family physicians [7, 53, 54], as are physicians practicing in the south [7]. Once we adjusted for interpersonal skills, we did not find that older physicians or males were more likely to prescribe antibiotics, associations that have been reported in a number of other studies [53, 55, 56].

Female physicians receive higher scores on OSCE examinations, primarily related to better communication skills [57, 58]. However, even after adjustment for interpersonal skills, female physicians were more likely to prescribe antibiotics. In exploratory analysis, we found that female physicians were more likely to prescribe antibiotics for patients presenting with sinusitis (OR: 1.46, 95% CI 1.28–1.67), not URI (OR: 1.10, 95% CI 0.99–1.24). Female physicians are more likely than male physicians to prescribe medication for pain [59–61]. Compared to URIs, patients with sinusitis are more likely to have considerable pain in relationship to their infection [62, 63], which may be why, on the off chance that it is a bacterial not a viral infection, female physicians are more likely to prescribe antibiotics. Another possibility is that patients are more likely to ask or demand antibiotics from female physicians, as there are differences in male and female patient behavior by physician gender [64–66, 59–63].

The citizenship of the international medical graduate at the time of their medical school education was significantly associatied with antibiotic prescribing, with the odds of prescribing being lower among citizens of all other countries compared to U.S. citizens, significantly so for Africa, South and Central America. Substantial differences in antibiotic prescribing by dentists [67]in the U.S, Canada, Australia and England has also been reported with US dentists being the highest [68]. Future research should focus on the role of culture and medical education in promulgation of unnecessary antibiotic prescribing.

Similar to prior studies, we found patients with sinusitis are more likely to receive antibiotics than patients with upper respiratory infections [5, 69, 70], as were white patients compared to black patients [6, 55] and patients with higher levels of co-morbidity [6, 7] when measured by the number of concurrent medications.

Our research has limitations that need to be considered in interpreting the results. First, we had no information on the clinical signs and symptoms that patients presented with at the time of the visit for sinusitis or upper respiratory infection with the study physicians. Both positive and worsening symptoms increase the risk of antibiotic prescribing [8]. While we do not expect there to be differences in the severity of a patient’s presentation in relationship to the physician’s clinical or communication skills, internal medicine specialists may see more symptomatic, co-morbid patients, which may explain the greater risk of antibiotic prescribing, although these differences, if any, appear to be modest [67]. We also had no measure of visit length or daily practice volume. Shorter visits and higher daily volume increase the risk of antibiotic prescribing [4, 56]. Physicians with better interpersonal skills may spend more time with patients, and as a result see fewer patients per day. This may be one plausible mechanism for why shorter visits and high volume increase the risk of antibiotic prescribing.

In conclusion, physicians who are international medical school graduates with better interpersonal skills are less likely to prescribe antibiotics to patients presenting with upper respiratory infection and acute sinusitis. Future research should examine whether tailored interpersonal skills training to help physicians manage patient expectations for antibiotics [45] during medical school or continuing professional education would reduce the risk of unnecessary antibiotic prescribing.

### Electronic supplementary material

Below is the link to the electronic supplementary material.

Supplementary Material 1

## Data Availability

The data analyzed in this study are not publicly available. Those wishing to obtain the data used in this study should contact Dr. John Boulet (bouletjr@gmail.com), the data custodian for the Educational Commission for Foreign Medical Graduates, as well as the Centre for Medicare & Medicaid Services DUA team (DataUseAgreement@cms.hhs.gov re: project number DUA RSCH-2017-51585) to obtain approval for access.
