# Supplementary material for: Do clinical and communication skills scores on credentialing exams predict potentially inappropriate antibiotic prescribing?
Source: BMC Med Educ. 2023 Nov 1;23:821. doi: 10.1186/s12909-023-04817-w (PMC10621187; doi:10.1186/s12909-023-04817-w)
Supplement: Supplementary file 1 — Supplementary Material 1 [file 12909_2023_4817_MOESM1_ESM.docx]

**Appendix 1. ICD codes for Upper Respiratory Infection and Sinusitis**

| **ICD9** | **ICD10** | **Definition** | **Condition** |
| --- | --- | --- | --- |
| 4610 | J0100 | AC MAXILLARY SINUSITIS | Sinusitis |
| 4611 | J0110 | AC FRONTAL SINUSITIS | Sinusitis |
| 4612 | J0120 | AC ETHMOIDAL SINUSITIS | Sinusitis |
| 4613 | J0130 | AC SPHENOIDAL SINUSITIS | Sinusitis |
| 4618 | J0140 | OTHER ACUTE SINUSITIS | Sinusitis |
| 4619 | J0190 | ACUTE SINUSITIS NOS | Sinusitis |
| 4650 | J060 | ACUTE LARYNGOPHARUNGITIS | Upper respiratory infection |
| 4658 | J069 | ACUTE URI MULT SITES NEC | Upper respiratory infection |
| 4659 |  | ACUTE URI NOS | Upper respiratory infection |
